# Purification of Peroxisomes in a Preformed Iodixanol Gradient in a Fixed-Angle Rotor

**DOI:** 10.1100/tsw.2002.838

**Published:** 2002-06-07

**Authors:** John Graham

**Affiliations:** School of Biomolecular Sciences, Liverpool John Moores University, UK

**Keywords:** peroxisomes, light mitochondrial fraction, OptiPrep™, iodixanol, liver, continuous gradient

## Abstract

In iodixanol, peroxisomes are the densest organelle in the light mitochondrial fraction and are therefore easily separated from the other components (lysosomes, mitochondria, etc.) in a preformed isosmotic continuous gradient. The resolution of the peroxisomes is far superior than that in sucrose and, unlike in Percoll® there is no contamination from endoplasmic reticulum.

**METHOD TYPE:** extraction, isolation, purification and separation

**SUB METHOD TYPE:** centrifugation

## INTRODUCTION

Peroxisomes can be purified in iodixanol gradients in high yield (80–90%) with no detectable contamination from any other organelle. This Protocol Article describes the use of a preformed continuous gradient in a fixed-angle rotor. Alternative strategies using either a simple high-density barrier in a swinging-bucket rotor[1] or a self-generated gradient[2] can be used with the same gradient medium.

In iodixanol, peroxisomes are the densest of the major subcellular organelles (ρ = 1.18–1.20 g/ml) present in the light mitochondrial fraction from mammalian tissues and cells. Mitochondria have a median density of approx 1.145 g/ml and lysosomes approx 1.115 g/ml. Metrizamide or Nycodenz® gradients have been used previously to purify peroxisomes[3,4] but because mitochondria have a higher density in Nycodenz® or metrizamide than in iodixanol, the resolution of these two organelles is much easier in iodixanol gradients[5,6,7]. In Percoll®, both peroxisomes and endoplasmic reticulum (ER) have the same banding density and these two organelles cannot be resolved; in iodixanol the ER has a much lower density.

The protocol below is for rat liver; it is adapted from the method of Van Veldhoven et al.[6]. Other tissues may require modified gradient conditions.

## MATERIALS AND EQUIPMENT

OptiPrep™
Homogenization medium (HM): 0.25 M sucrose, 1mM EDTA, 0.1% (v/v) ethanol, 5 mM Mops pH 7.2
Diluent: 6 mM EDTA, 0.6% ethanol, 30 mM Mops, pH 7.2
1 M sucrose

High-speed centrifuge with a fixed-angle rotor (30- to 50-ml tubes)
Ultracentrifuge with a fixed-angle rotor (30- to 50-ml tubes) capable of approx. 100,000*g*
Potter-Elvehjem homogenizer (30–40 ml), clearance approx 0.08 mm
Wall-mounted, high-torque, thyristor-controlled electric motor
Dounce homogenizer (10 ml, loose-fitting, Wheaton Type B)
Gradient Master or two-chamber gradient maker
Gradient unloader (tube puncture)
Syringe and metal cannula (for underlayering)

## METHOD

Carry out all operations at 0–4°C.

1. Make up the following gradient solutions from OptiPrep™, Diluent, 1 M sucrose, and water using, respectively, these ratios by volume (see Note 1):
   1: 5 + 0.6 + 0.4 + 0 (50% iodixanol)
   2: 4 + 0.6 + 0.7 + 0.7 (40% iodixanol)
   3: 2 + 0.6 + 1.1 + 2.3 (20% iodixanol)
2. Mince the liver very finely with scissors and transfer to a Potter-Elvehjem homogenizer with HM (use 10 ml medium for every 2.5 g tissue). Homogenize using approx. 6 strokes of the pestle (500–700 rpm) (see Note 2).
3. Centrifuge the homogenate at $3000g_{av}$ in a fixed-angle rotor for 10 min to pellet the nuclei and heavy mitochondria. This pellet may be rehomogenized in HM and the centrifugation repeated.
4. Centrifuge the supernatant(s) at $17{,}000g_{av}$ for 10–15 min.
5. Resuspend the 17,000*g* pellet in HM using a loose-fitting Dounce homogenizer (2–3 strokes of the pestle). Adjust to a volume of about 0.5 ml/g of tissue.
6. Use a two chamber gradient maker or a Gradient Master to prepare a linear gradient from 9 ml each of gradient solutions 2 and 3 in thick-walled polycarbonate tubes for a 36–40 ml fixed-angle rotor and underlayer each gradient with 2 ml of gradient solution 1 (see Note 3).
7. Layer 3 ml of the suspension over each gradient and centrifuge at $105{,}000g_{av}$ for 1 h.
8. Allow the rotor to decelerate from 1,000 rpm without the brake, the collect the gradient in 1 ml fractions dense end first (see Note 4).

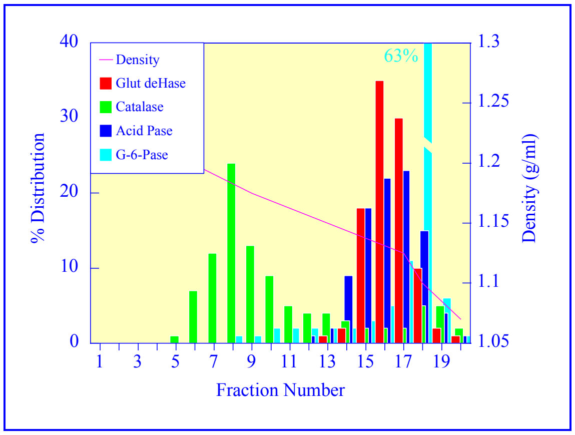

**FIGURE 1**. Isolation of peroxisomes: enzyme distribution in gradient. Glut deHase = glutamate dehydrogenase, Acid Pase = acid phosphatase; G-6-Pase = glucose-6-phosphatase. Adapted from Ref. [4] with kind permission of Academic Press and the authors.

## ANALYSIS

Iodixanol does not significantly inhibit any enzyme so far tested. Standard spectrophotometric methods (carried out above 340 nm), for measuring organelle enzyme markers can be performed directly on gradient fractions[8]. Protein can also be measured directly by any Coomassie blue-based method[8]. If it is necessary to remove the gradient medium, dilute fractions with an equal volume of buffer; pellet at approx. 30,000$g_{av}$ for 10 min and resuspend in a suitable buffer.

A typical result is shown in Fig. 1, which shows the distribution of marker enzymes across the gradient. The catalase (peroxisome) band is well separated from all of the mitochondria (GDH) and lysosomes (acid phosphatase). The endoplasmic reticulum (glucose-6-phosphatase) is the least dense membrane type. This is in contradistinction to Percoll® gradients (see Note 5) in which ER and peroxisomes always overlap.

## NOTES

1. The variable volume of 1 M sucrose maintains each solution isoosmotic. Keep these solutions, and carry out all subsequent operations, at 0–4°C. Protease inhibitors may be included in any or all of the media at the operator's discretion.
2. See Refs. [9,10,11], respectively, for more information on homogenization of tissues and cells and differential centrifugation of a homogenate.
3. Thin-walled tubes can be used but may require some capping or sealing device. See Ref. [12] for more details on the preparation of preformed iodixanol gradients.
4. Gradients can be unloaded dense end first by carefully introducing a narrow metal cannula (connected to a peristaltic pump) to the bottom of the tube. Thin-walled tubes can be collected by tube puncture. For more information see Ref. [13].
5. Yields of peroxisomes from Percoll® gradients are often low due to loss of material during the final centrifugation step to remove the Percoll®. This may be necessary if the light scattering properties of Percoll® interfere with any subsequent spectrophotometric or SDS-PAGE analyses.

## ACKNOWLEDGEMENTS

The author and TheScientificWorld wish to thank Axis-Shield PoC, AS, Oslo, Norway for their kind permission to adapt OptiPrep™ Application Sheet S9 in the preparation of this Protocol Article.
